# Standardized Reporting in Gynecological Ultrasonography in India: The Need of the Hour

**DOI:** 10.7759/cureus.115562

**Published:** 2026-08-31

**Authors:** Gaurav Khastgir, Sujata Siwatch

**Affiliations:** 1 Obstetrics and Gynecology, Bengal Infertility & Reproductive Therapy Hospital (BIRTH), Kolkata, IND; 2 Obstetrics and Gynecology, Post Graduate Institute of Medical Education and Research, Chandigarh, IND

**Keywords:** adenomyosis, adnexal masses, diagnostic imaging, endometriosis, gynaecology, iota-adnex model, iota simple rules, o-rads ultrasound, standardisation, ultrasonography

## Abstract

Gynecological ultrasonography (USG) is integral to evaluating adnexal masses, adenomyosis, endometriosis, and other pelvic pathologies. Despite advances in imaging, substantial variability in terminology and reporting persists, potentially leading to inconsistent communication and clinical decision-making. Established frameworks, including the Ovarian-Adnexal Reporting and Data System (O-RADS) and International Ovarian Tumor Analysis (IOTA) Simple Rules (SR) for structured assessment of adnexal masses, the Assessment of Different NEoplasias in the adneXa (ADNEX) model for risk prediction, Morphological Uterus Sonographic Assessment (MUSA) terminology for adenomyosis, and International Federation of Gynecology and Obstetrics (FIGO) classifications, serve complementary roles in description, diagnosis, risk stratification, and classification.

Standardized approaches may improve diagnostic consistency and facilitate clinical communication, research, audit, education, and the development of artificial intelligence-based tools. However, implementation requires appropriate training, familiarity with standardized terminology, and integration into routine clinical workflows. Drawing parallels with the widespread adoption of the Breast Imaging Reporting and Data System (BI-RADS) in breast imaging, this commentary highlights the importance of broader adoption of standardized terminology and structured reporting in gynecological ultrasonography. Greater engagement of national professional societies, aligned with established international frameworks and recommendations from the International Society of Ultrasound in Obstetrics and Gynecology (ISUOG), may help promote more consistent reporting practices and enhance patient care.

## Editorial

The landscape of gynecological imaging has undergone a remarkable transformation over the past few decades. Ultrasonography (USG) now plays a central role in diagnosing a wide spectrum of gynecological disorders, including adnexal masses, endometriosis, adenomyosis, uterine fibroids, congenital anomalies, and placenta accreta spectrum. Higher imaging modalities such as Computed Tomography (CT) and Magnetic Resonance Imaging (MRI) are also used to characterize lesions in detail, identify their origin, and rule out distant spread, especially in malignancies. Despite these advances, significant variability persists in imaging terminology, reporting practices, and interpretation across healthcare settings [1].

USG remains the preferred initial imaging choice because of its cost-effectiveness and availability. However, variability in structured evaluation, operator experience, interpretation, and communication between radiologists and clinicians may contribute to differences in real-world diagnostic performance. The lack of standardized terminology, particularly in gynecological radiology, may further contribute to miscommunication and potentially delay appropriate diagnosis and management. Variations in assessment are influenced by both clinical factors (study population, cancer prevalence, and classification) and technical factors (equipment and expertise). The American College of Radiology (ACR) has introduced standardized reporting systems to improve communication and guide clinical decisions across different organ pathologies. Similarly, efforts to standardize reporting in gynecology have led various international bodies to introduce lexicons, risk prediction models, classification systems, and diagnostic rules aimed at enhancing communication between sonologists and physicians and reducing variability in reporting. 

Timmerman et al. proposed a set of standardized terminologies and guidelines for use in future studies. This heralded the evolution of a set of standardized terminology that is used by the International Ovarian Tumor Analysis Group (IOTA) Simple Rules and Assessment of Different NEoplasias in the adneXa (ADNEX) risk stratification model to differentiate between benign and malignant adnexal masses [2]. In 2015, the Ovarian-Adnexal Reporting and Data System (O-RADS) Committee was established under ACR supervision to reduce ambiguity in ultrasound reports. The O-RADS model provides a comprehensive lexicon that categorizes adnexal masses from normal (O-RADS 1) to high risk of malignancy (O-RADS 5).

Consistent terminology allows better comparison of data between institutions, enhancing both clinical research and audit processes. Additionally, structured reports help train residents and fellows by fostering a shared language and minimizing subjective interpretation. Structured reporting also establishes a solid basis for creating artificial intelligence-based diagnostic tools, which require uniform imaging descriptors and well-organized datasets for effective algorithm training and validation. With a large and expanding diagnostic imaging ecosystem, a diverse patient population, and substantial clinical workloads across healthcare settings, India is well positioned to generate high-quality multicentric datasets that can inform future research and guideline development.

A striking example of the benefits of standardization is evident in breast imaging. Today, mammography and breast ultrasound reports widely use a Breast Imaging Reporting and Data System (BI-RADS) category, enabling clear communication and facilitating uniform management decisions. In contrast, based on our observations of current clinical practice, despite the availability of validated systems such as O-RADS and the ADNEX model, their incorporation into routine reporting of adnexal masses in India appears to remain variable. Similarly, although some sonologists may describe the International Federation of Gynecology and Obstetrics (FIGO) classification of uterine myomas, routine use of the Morphological Uterus Sonographic Assessment (MUSA) terminology for the standardized sonographic assessment of adenomyosis appears less established. As a result, reports often remain descriptive, with significant inter-observer variability and ambiguity.

A recent prospective study compared the diagnostic accuracies of O-RADS, International Ovarian Tumor Analysis Group Simple Rules (IOTA SR), ADNEX, and Risk of Malignancy Index (RMI 4) in the Indian population. O-RADS had the highest sensitivity (98%), followed by IOTA SR (95%), RMI 4 (88%), and ADNEX (85%). ADNEX showed the highest specificity (95%), followed by RMI 4 (89%), IOTA SR (87%), and O-RADS (53%). O-RADS demonstrated excellent sensitivity in both pre- and postmenopausal women, accurately predicting all malignant epithelial lesions. The ADNEX model consistently outperformed the other systems in identifying true negatives and had the best specificities across age groups and histopathologies [3]. A study has also demonstrated the diagnostic performance of the ADNEX model at various cut-offs in an Indian cohort, describing a practical approach of using this model across various clinical settings. Lower thresholds (5-20%) demonstrated high sensitivity but poor specificity, whereas higher thresholds (40-50%) improved specificity and positive predictive values at the expense of sensitivity. A 25-30% threshold provided a favorable balance between sensitivity and specificity, while the Youden index statistically identified optimal cut-offs around 43% [4]. Another prospective observational study from Eastern India found significant associations between specific direct and indirect MUSA features and symptom severity. Heavy menstrual bleeding was associated with asymmetrical myometrial thickening, interrupted junctional zone, and globular uterus, while myometrial cysts were associated with chronic pelvic pain and dysmenorrhoea [5].

Greater adoption of such reporting strategies would provide a common language for radiologists and gynecologists, improve risk stratification, and support evidence-based decision-making. Achieving this will require collaboration among trained gynecologists, radiologists, sonologists, and relevant professional societies, with a shared commitment to incorporating standardized terminology and reporting systems into routine practice. Such a collaborative approach would promote uniformity in reporting, improve interdisciplinary communication, and ultimately enhance patient care. Practical implementation could begin with developing and disseminating standardized reporting templates based on existing international guidelines, supported by targeted training programs. Pilot implementation across selected centers, followed by periodic quality audits and feedback, could help identify barriers and refine the process before wider adoption. 

We hope to see a future where standardized gynecological radiological reporting becomes routine practice across India, much like BI-RADS, which has become integral to breast imaging. A coordinated national initiative involving the Federation of Obstetric and Gynecological Societies of India (FOGSI) and the Indian Radiological and Imaging Association (IRIA) to promote structured reporting in accordance with the International Society of Ultrasound in Obstetrics & Gynecology (ISUOG) guidelines may be a timely and important step. 
